# A Dual-Action Gold(I) Prodrug Targeting Redox Homeostasis and Extracellular Matrix Remodeling in Ovarian Cancer

**DOI:** 10.1021/acsmedchemlett.6c00118

**Published:** 2026-06-08

**Authors:** Riccardo Di Leo, Enrico Crispino, Lorenzo Chiaverini, Luca Famlonga, Rosamaria Militello, Tania Gamberi, Iogann Tolbatov, Alessandro Marrone, Diego La Mendola, Tiziano Marzo, Elisa Nuti

**Affiliations:** † Department of Pharmacy, 9310 University of Pisa, via Bonanno 6, 56126 Pisa, Italy; ‡ Institute of Clinical Physiology, 117064 National Research Council (CNR), via Moruzzi 1, 56124 Pisa, Italy; § Department of Experimental and Clinical Biomedical Sciences “Mario Serio”, 9300 University of Florence, Viale GB Morgagni 50, 50134 Florence, Italy; ∥ Department of Chemical, Physical, Mathematical and Natural Sciences, 9312 University of Sassari, 07100 Sassari, Italy; ⊥ Department of Pharmacy, University “G. D’Annunzio”, via dei Vestini 31, 66100 Chieti, Italy

**Keywords:** ovarian cancer, matrix metalloproteinases (MMPs), auranofin, gold compounds, thioredoxin reductase

## Abstract

Ovarian cancer is characterized by early metastatic dissemination, frequent relapses, and limited therapeutic options following the onset of platinum resistance. To address these challenges, a dual-action gold­(I) prodrug, RDL-15, was designed to concurrently interfere with intracellular redox regulation and extracellular matrix remodeling. RDL-15 combines the gold­(I) pharmacophore derived from Auranofin with the scaffold of LP-158, a carboxylate-based inhibitor of gelatinases MMP-2 and MMP-9 previously described. The complex retains micromolar cytotoxic activity in ovarian cancer cell lines A2780 and SKOV-3, including the cisplatin and AF-resistant A2780/R variant, and induces an early reduction of thioredoxin reductase activity. In SKOV-3 cells, characterized by high migratory and invasive capacity, RDL-15 significantly suppresses migration and invasion, whereas Et_3_PAuCl alone shows no intrinsic anti-invasive effect. Computational analyses further support a structure–function relationship linking gold–ligand bonding features to the observed biological profile.

## Graphical abstract

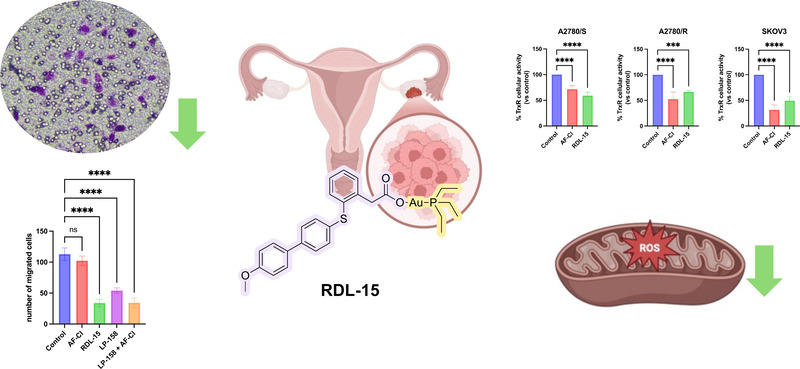

Ovarian cancer (OC) is among the most common gynecological malignancies, together with cervical and uterine cancers, yet it remains the most aggressive and lethal.^1^ Its clinical burden is driven largely by delayed diagnosis, early metastatic dissemination and frequent development of resistance to therapy, especially in the case of relapse. Because early stage disease is often clinically silent and reliable markers for early detection are lacking, approximately 70–80% of patients are diagnosed only after the tumor has reached advanced stages, often resulting in poor clinical outcomes.^2,3^ This context is further influenced by the biological features and therapeutic management of the predominant tumor subtype. More than 90% of ovarian cancers emerge from epithelial tissues and are classified as epithelial ovarian cancer (EOC).^4^ EOC, is a biologically heterogeneous disease, typically treated with cytoreductive surgery followed by platinum-based chemotherapy.^5^ Although this approach often induces an initial tumor regression, microscopic residual disease commonly persists, leading to relapse.^6^ At the molecular level, platinum compounds induce cytotoxicity effects mainly through DNA cross-linking, but also through mitochondrial dysfunction, oxidative stress, and membrane perturbations, imposing strong selective pressure.^7–9^ In fact, some tumor cells are able to survive after this treatment due to both their DNA repair capacity and their reinforced antioxidant defenses, enabling them to tolerate sustained genotoxic stress and promoting the emergence of platinum resistance. In parallel, EOC cells display pronounced metabolic plasticity. They can depend on aerobic glycolysis as well as mitochondrial oxidative phosphorylation to support proliferation, invasion, and metastatic dissemination. The relative contribution of these pathways shifts according to tumor subtype and the surrounding microenvironment. Such metabolic adaptability may contribute to therapeutic resistance.^6^ Accordingly, therapeutic options for metastatic OC remain limited, particularly after the onset of resistance. Thus, although several new chemotherapeutic agents have been introduced, their clinical impact remains modest owing to high cost, limited accessibility, and clinical outcomes that do not substantially improve upon conventional therapies. These constraints have prompted interest in alternative approaches that act through mechanisms distinct from conventional cytotoxic drugs, including nonplatinum metal-based compounds such as gold-containing agents, which target redox-sensitive cellular processes.^10^ Among these approaches, gold­(I) complexes have attracted significant attention, given their prior clinical application and their unique mode of action driven by redox modulation. Auranofin [2,3,4,6-tetra-o-acetyl-*L*-thio-β-d-glyco-pyranosato-*S*-(triethyl-phosphine)-gold­(I)] (AF) is a gold­(I) compound (Figure 1) with long-standing clinical use, originally developed as a therapeutic agent for the long-term treatment of rheumatoid arthritis. Beyond its anti-inflammatory activity, AF has attracted renewed interest as a candidate for drug repurposing in oncology, supported by extensive preclinical evidence of antiproliferative activity across multiple tumor models and by its ongoing evaluation in clinical trials for solid and hematological malignancies, including ovarian cancer.^11^ Mechanistically, AF exerts its biological effects through the modulation of redox-sensitive pathways, most notably via inhibition of thioredoxin reductase (TrxR), disruption of mitochondrial function, and induction of oxidative stress, processes that overlap with, yet are mechanistically distinct from, those targeted by platinum-based chemotherapy.^12–14^ Structural and bioinorganic studies have established that the biological activity of AF resides primarily in the invariant [Et_3_PAu]^+^ moiety. In fact, this cationic fragment, which generates upon release of the thiosugar ligand, acts as the true pharmacophore, whereas the thiosugar ligand plays a secondary role, mainly acting as a labile leaving group.^15,16^ In line with this concept, AF analogues in which the thiosugar is replaced by alternative ligands, including halides,^17,18^ retain significant biological activity while offering the possibility to finely tune physicochemical properties such as lipophilicity, bioavailability, and biodistribution. These observations provide a strong rationale for the development of gold­(I) complexes preserving the AF pharmacophore while selectively modifying the sugar-derived moiety to optimize anticancer efficacy. Alongside the role of gold compounds, extracellular matrix (ECM) remodeling has emerged as a central driver of OC progression and metastatic dissemination.^19^ OC cells must breach the mesothelial cell layer and invade the submesothelial ECM to establish peritoneal metastases, a process tightly regulated by proteolytic enzymes. Among these, matrix metalloproteinases (MMPs) play a pivotal role by coordinating ECM degradation, cell migration, invasion, inflammation, and angiogenesis.^20,21^ It is noteworthy that gelatinases MMP-2 and MMP-9 have been consistently implicated in OC aggressiveness.^22^ These enzymes degrade type IV collagen, the main component of the basement membrane, removing a critical physical barrier to invasion.^23^ Furthermore, the cleavage of fibronectin and vitronectin, mediated by MMP-2, generates proteolytic fragments with enhanced adhesive properties, facilitating tumor cell attachment to mesothelial surfaces during early metastatic seeding.^24^ Similarly, MMP-9 not only degrades ECM components but also promotes epithelial–mesenchymal transition through the release and activation of transforming growth factor-β, further amplifying invasive behavior.^25,26^ The activity of MMP-2 is regulated by membrane-type 1 MMP (MT1-MMP/MMP-14). MT1-MMP/MMP-14 is overexpressed in OC relative to normal and benign ovarian tissue and correlates with poor prognosis and enhanced metastatic potential. Together, dysregulated MMP-2/9 activity integrates mechanical ECM remodeling with pro-migratory and pro-invasive signaling, positioning these proteases as key molecular effectors of OC dissemination and attractive targets for therapeutic intervention. Moreover, other MMPs contribute through complementary mechanisms that facilitate efficient extracellular matrix penetration and the metastatic anchoring of OC cells.^23^

**Figure 1:**
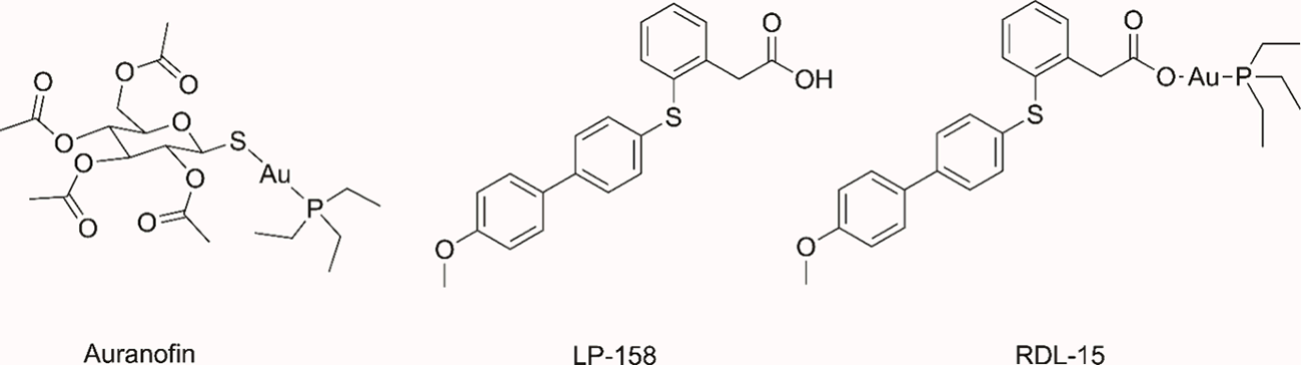
Chemical structures of Auranofin, LP-158, and RDL-15

On this basis, a dual-action prodrug was designed to target redox dysregulation and extracellular matrix remodeling in OC. The system was designed to release the gold­(I) pharmacophore of AF together with an inhibitor of the gelatinases MMP-2/9. This design exploits the [Et_3_PAu]^+^ moiety as the primary determinant of AF cytotoxic activity, enabling modification of the coordinated ligand environment. Accordingly, the thiosugar was replaced through conjugation with LP-158 (Figure 1), a previously reported carboxylate-based MMP inhibitor displaying sub-μM potency toward MMP-2 and MMP-9 (IC_50–MMP‑2_ = 114 nM, IC_50–MMP‑9_ = 830 nM).^27^ This compound was chosen among others developed by Nuti et al. in consideration of its nanomolar inhibitory activity, chemical stability, and for the presence of a zinc-binding group, such as a carboxylic acid, which is also capable of coordinating gold­(I). Integration of these elements via an O–Au–P linkage couples redox stress induction with inhibition of ECM degradation and invasion, targeting both tumor survival and metastatic dissemination in OC cells.

The dual-action prodrug, RDL-15 (Figure 1, Scheme 1), was obtained via a gold–silver transmetalation route^15^ starting from Et_3_PAuCl and the silver salt of the carboxylic acid LP-158, synthesized as previously described.^27^ The silver intermediate was generated from the corresponding sodium salt by treatment with silver nitrate (AgNO_3_) under light-protected conditions.

**Scheme 1:**
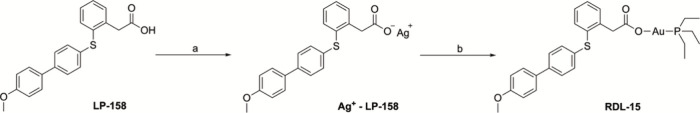
Reagents and Conditions: (a) AgNO_3_, NaOH, H_2_O, RT, 1 h, 91%; (b) Et_3_PAuCl, EtOH, RT, 3 h, 30%

Coordination of the carboxylate ligand to the [AuPEt_3_]^+^ fragment was evidenced by a marked upfield shift in the ^31^P NMR signal of the triethylphosphine ligand (27.5 ppm) relative to the reported Et_3_PAuCl (∼34 ppm in DMSO-*d*_6,_),^15,28,29^ this shift being consistent with literature reports for similar Au­(I) carboxylate complexes. Moreover, the ^1^H NMR spectrum displayed the characteristic multiplet signals associated with the CH_2_ and CH_3_ groups of the triethylphosphine ligand coordinated to Au­(I), appearing at 1.86 and 1.05 ppm, respectively. Consistently, the corresponding ^13^C NMR spectrum exhibited resonances at 17 and 9 ppm, attributable to the same alkyl moieties. The identity of the target compound was confirmed by high-resolution ESI-MS measurements (SI). Additionally, the elemental analysis closely matched the theoretical values, further confirming the chemical integrity and purity of the product.

The solution stability of RDL-15 was assessed at 37 °C by UV–Vis spectroscopy and ^31^P NMR in aqueous mixed media (UV–Vis in DMSO/PBS 1:1; NMR in DMSO-*d*_6_/D_2_O 1:1, Figures S1 and S2) as well as in plasma (in the presence of 5% of DMSO, Figures S3). In the ^31^P NMR spectrum in the DMSO/PBS mixture, the signal of the intact complex at 27.5 ppm was already absent at the initial time point, indicating a rapid transformation in solution under the specific conditions required for NMR spectroscopy (4.3 × 10^–3^ M). This process is marked by the appearance of two new resonances at 34.3 and 47.5 ppm, attributable to the formation of Et_3_PAuCl, together with a scrambling product corresponding to [Au­(PEt_3_)_2_]^+^ (SI). The signal assigned to [Au­(PEt_3_)_2_]^+^ is consistent with the well-known ligand scrambling behavior of Au­(I)–phosphine complexes previously reported for AF-derived compounds.^18^ From a pharmacological standpoint, this species is expected to be poorly relevant under biological conditions, where the presence of multiple high-affinity biomolecular partners inhibits its formation, which is instead favored under cell-free NMR conditions.^18^ Speciation is also supported by UV–Vis spectroscopy, which reveals a slower, time-dependent increase in absorption in the 270–310 nm region. This trend differs from what is observed by NMR, where at higher concentration (4.3 × 10^–3^ M) the RDL-15 complex breaks down immediately. At the lower concentration used for UV–Vis measurements (1.0 × 10^–4^ M), the process evolves more gradually, indicating that the transformation is not instantaneous but proceeds over time. Notably, the 270–310 nm region is dominated by the aliphatic features previously assigned, whose growth mirrors the evolution observed by NMR. Collectively, the solution speciation of RDL-15 under aqueous conditions supports a mechanism in which the Au­(I) center evolves toward the pharmacologically relevant auranofin-like species, while concomitantly enabling the release of the LP-158 fragment. This behavior is consistent with the molecular design of the system and provides a chemical basis for the simultaneous engagement of redox-sensitive intracellular targets and MMP-driven extracellular processes implicated in ovarian cancer progression. The same trend was independently confirmed in plasma using ^31^P NMR at a concentration of 5.4 × 10^–3^ M. In these experiments, the lock signal was directly obtained from blood plasma.^30^ Spectra indicate the formation of stable Au­(I)-containing bioconjugates under these biologically relevant conditions. Two major resonances at approximately 38.5 and 31.2 ppm were consistently observed. The downfield signal is compatible with a coordinated Au­(I)-triethylphosphine species interacting with plasma biomolecules, possibly including thiol-containing proteins such as albumin.^31–33^ After 48 h, a signal at approximately 47 ppm became more evident, plausibly reflecting slower ligand scrambling processes previously described for auranofin-related systems.^31^ Overall, the plasma behavior of RDL-15 closely resembles that reported for Au­(I)-phosphine derivatives of AF.^33^

RDL-15 was subsequently evaluated for its biological activity in comparison with LP-158 and Et_3_PAuCl (hereafter AF-Cl). AF-Cl has already been reported to display biological effects comparable to those of AF.^18^ The cytotoxic activity of the RDL-15 complex was assayed by MTT assay in cisplatin-sensitive A2780/S, cisplatin-resistant A2780/R, auranofin-resistant A2780/AF-R and SKOV-3 human ovarian cancer cell lines using cisplatin and auranofin as reference standard drugs (Table 1). RDL-15 showed a reproducible cytotoxic activity in the low micromolar range in all OC cell models analyzed, including the cisplatin-resistant A2780/R line (IC_50_ = 4.9 μM). These findings indicate that its anticancer activity is substantially preserved even in a platinum-resistant setting.

**Table 1:** Half-Maximal Inhibitory Concentration (IC_50_) of Et_3_PAuCl (AF-Cl), RDL-15 and LP-158 after 72 h of Treatment Using MTT Assay on OC Cells^a^

	Cell line – IC_50_ (μM) ± SD^a^				
Compound	**A2780/S**	**A2780/R**	**SKOV-3**	**A2780/AF-R**	**HEK293**
AF-Cl	3.7 ± 0.9	8.5 ± 1.8	5.7 ± 0.6	28.4 ± 1.1	14.5 ± 0.1
RDL-15	5.5 ± 0.1	4.9 ± 0.9	6.7 ± 1.5	16.7 ± 3.0	24.5 ± 0.6
LP-158	>100	>100	>100	>100	>100
Cisplatin	2.1 ± 0.2 ^35^	24.4 ± 0.1 ^36^	3.6 ± 0.8 ^37^	32.6 ± 5.5	19.6 ± 2.3 ^38^
AF	0.7 ± 0.1	3.9 ± 0.3	2.6 ± 0.4	7.2 ± 0.1	13.8 ± 0.5

a Auranofin and cisplatin were used as control drugs.

b Values are expressed as mean ± standard deviation (SD) of three biological independent experiments

In detail, AF-Cl displayed a more variable response across cell lines, with a slight decrease in efficacy in resistant cells. In sharp contrast, LP-158 alone did not interfere with cell viability, highlighting that in these systems the inhibition of MMP activity is not associated with intrinsic antiproliferative effects. Hence, these results are in line with our dual-drug design, in which the incorporation of LP-158 into the gold­(I) scaffold retains the cytotoxic contribution of the Au–phosphine core while maintaining activity in platinum-resistant OC cells.

To better elucidate the contribution of the gold-containing pharmacophore versus that of the organic scaffold, the compounds were also screened against the A2780/AF-R line. Interestingly, while AF-Cl and AF showed a marked drop in efficacy due to gold-specific resistance mechanisms, RDL-15 largely retained its antiproliferative effects (Table 1). These data suggest that the structural integration of the LP-158 moiety successfully attenuates or bypasses the standard gold-resistance pathways. Additionally, cytotoxicity was evaluated on the noncancerous human embryonic kidney cell HEK293 to assess general toxicity. RDL-15 was significantly less toxic to healthy cells than to cancer cells, highlighting a promising safety profile for this novel complex (Table 1).

Subsequently, the TrxR activity of RDL-15 was investigated 24 h after treatment at concentrations corresponding to the IC_50_ values determined at 72 h (Figure 2). Basal TrxR activity was higher in cisplatin-resistant A2780/R cells and in SKOV-3 cells than that in cisplatin-sensitive A2780 cells. This points to intrinsic differences in redox-related enzyme levels across the ovarian cancer models examined.^34^ Upon exposure to AF-Cl or RDL-15, TrxR activity was reduced in all cell lines with respect to the control, indicating early interaction with the thioredoxin system. Importantly, RDL-15 decreased TrxR activity also in cell lines characterized by elevated basal enzyme levels and showed a slightly higher effect on A2780/S cells with respect to that of AF-Cl alone.

**Figure 2:**
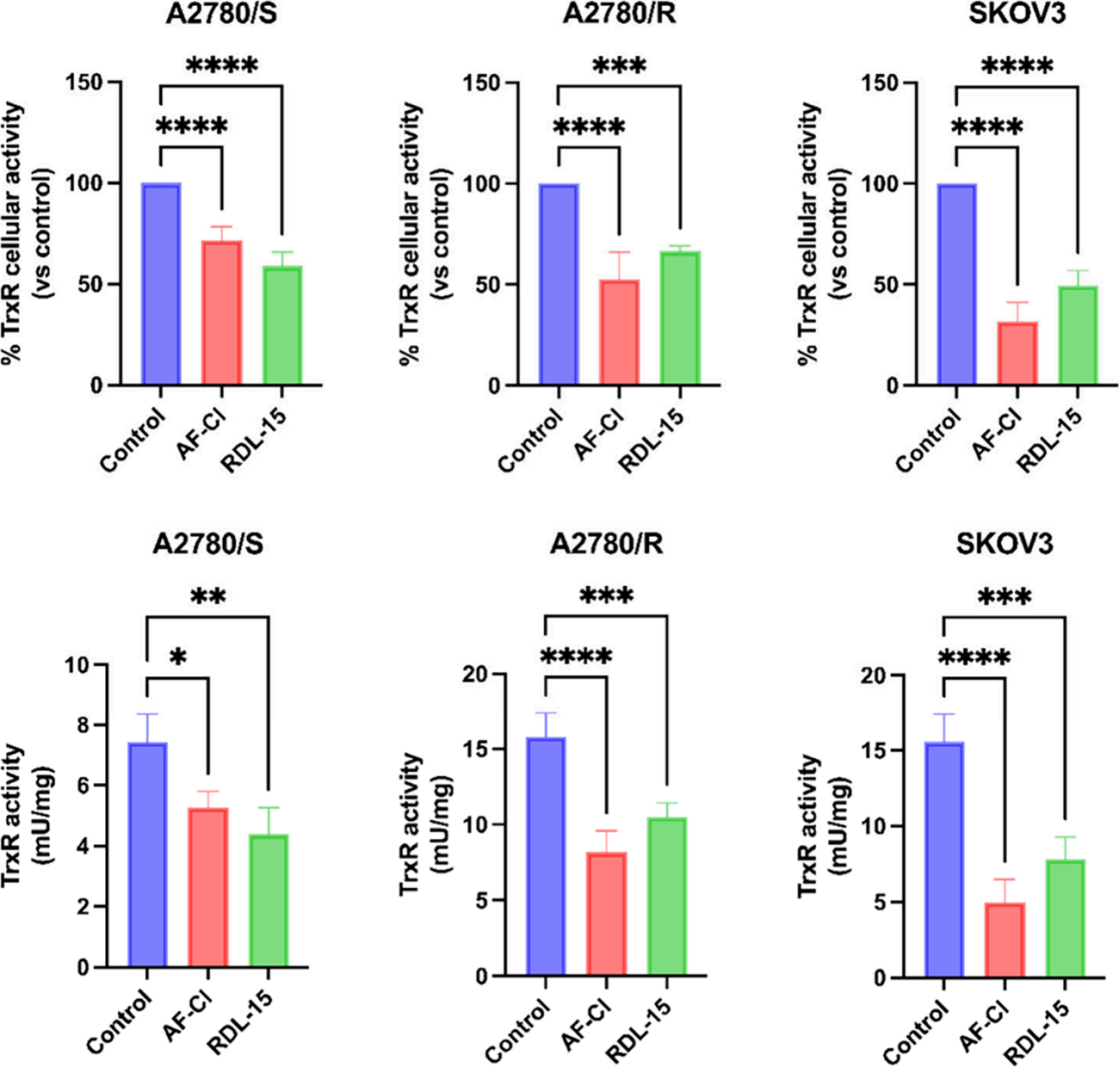
Thioredoxin reductase (TrxR) activity in A2780/S, A2780/R, and SKOV3 cell lines, treated for 24 h with AF-Cl and RDL-15 concentrations corresponding to their 72 h-exposure IC_50_ doses. Results are reported as both a percentage of TrxR activity versus control and as mU/mg. The statistical analysis was carried out using one-way ANOVA test followed by Tukey’s multiple comparisons test using GraphPad Prism software v 6.0 (*p < 0.05; ** *p* < 0.01; *** *p* < 0.001, **** *p* < 0.0001).

At this point, considering that LP-158 does not display intrinsic cytotoxic activity, its contribution to the biological profile of RDL-15 was further evaluated by examining its ability to inhibit MMPs implicated in OC invasion. The newly synthesized complex RDL-15 was tested *in vitro* on human recombinant MMP-2 and MMP-9 by a fluorometric assay in comparison with LP-158 and AF-Cl alone and with a coadministration of LP-158/AF-Cl. Alongside the reported inhibitory activity of LP-158,^27^ the gold­(I) precursor AF-Cl showed no inhibitory activity against MMP-2 and MMP-9 in an *in vitro* enzymatic assay. Noteworthy, conjugation of LP-158 to the gold­(I) scaffold in RDL-15 moderately enhanced gelatinase inhibition (MMP-2, IC_50_ = 73 nM), indicating that coordination to the [AuPEt_3_]^+^ moiety does not compromise MMP-targeting activity of LP-158 (Table 2). As expected, an effect similar to RDL-15 on gelatinase inhibition was evidenced for the coadministration of LP-158 and AF-Cl. Both the parent compound LP-158 and the gold complex RDL-15 showed a 7-fold stronger inhibitory activity against MMP-2 than on MMP-9. This effect can be explained considering the different depth of the S1’ “selectivity pocket”,^39^ situated near the catalytic zinc ion of MMPs. This pocket is the most variable one in the catalytic site of MMPs, has a deeper shape in MMP-2 than in MMP-9 and MMP-2 can better accommodate the *p*-methoxybiphenyl moiety of LP-158 and RDL-15, as previously shown for similar classes of inhibitors.^40,41^

**Table 2:** *In Vitro* ^a^ Inhibitory Activity (IC_50_, nM) on MMPs by a Fluorometric Assay

Compound	MMP-2	MMP-9
LP-158	114 ± 5.3 ^27^	830 ± 79 ^27^
RDL-15	73 ± 2	575 ± 15
AF-Cl	>100000	>100000
LP-158 + AF-Cl	68 ± 3	806 ± 44

a Assays were performed in duplicate. Values are reported as mean ± SD.

Finally, to assess whether enzymatic inhibition translated into functional effects at the cellular level, migration and invasion assays were conducted in SKOV-3 cells, which display higher migratory and invasive properties than A2780 cells (Figure 3). Treatment with LP-158 at 10 μM concentration significantly reduced both migratory and invasive behavior compared with untreated controls, whereas AF-Cl alone did not affect these processes. Importantly, RDL-15 (at 6.7 μM) suppressed cell migration and invasion slightly more effectively than LP-158, consistent with effective inhibition of gelatinase-dependent matrix remodeling. The coadministration of LP-158 and AF-Cl reproduced a similar inhibitory effect, supporting the contribution of the MMP-inhibitory component to the anti-invasive activity observed. Together, these findings indicate that incorporation of LP-158 into RDL-15 confers functional suppression of OC cell migration and invasion, complementing the redox-mediated cytotoxic effects of the gold­(I) pharmacophore.

**Figure 3:**
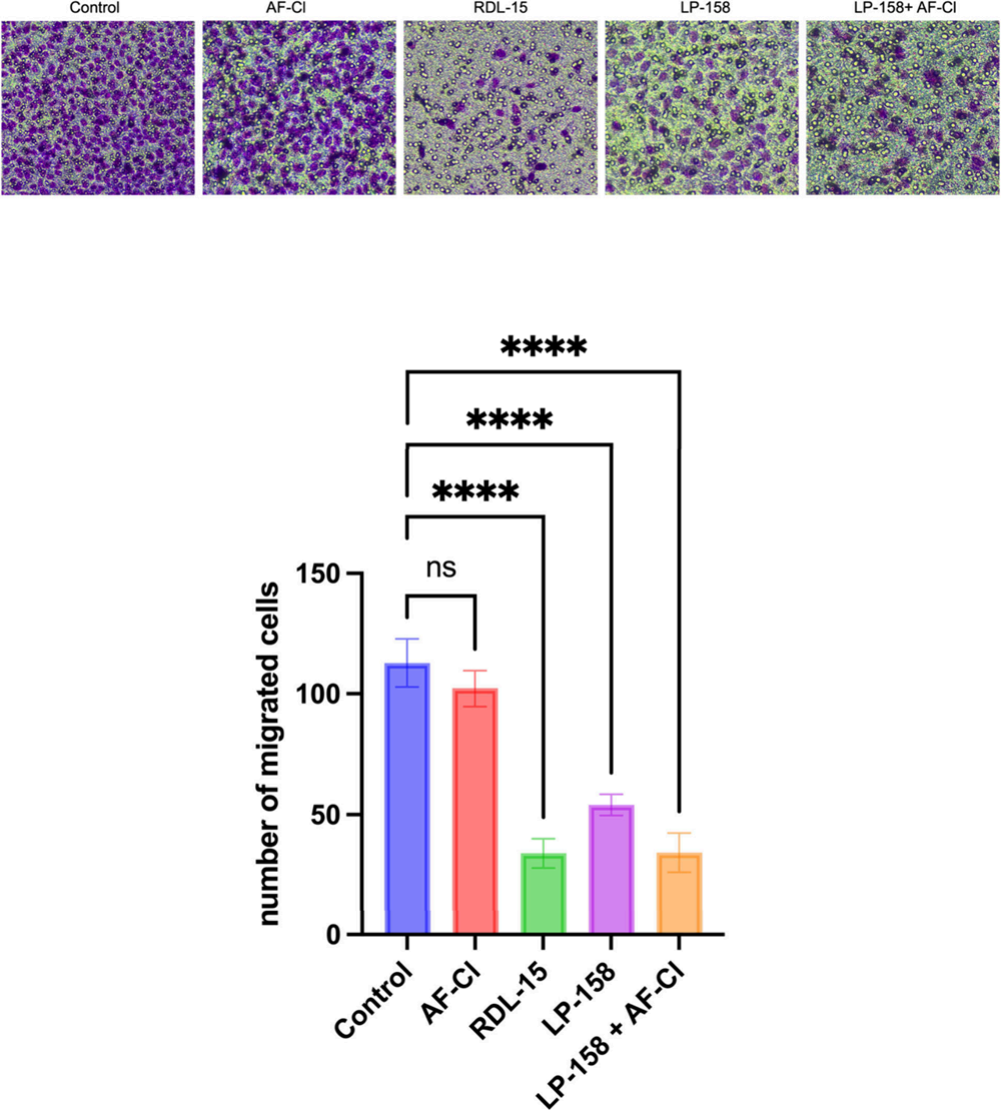
Cell migration and invasion was determined on trans-well inserts precoated with 50 μg/cm^2^ Matrigel. Images are representative. The percentage of wound reduction was determined using ImageJ software. Graphs reports mean ± SD obtained in three independent experiments. The statistical analysis was carried out using one-way ANOVA test followed by Tuckey’s multiple comparisons test using GraphPad Prism v 6.0 (****p < 0.0001).

To gain molecular insight into the distinct biological behaviors observed for AF-Cl and RDL-15, a comparative computational analysis was carried out to assess the strength and electronic features of the Au–ligand bonds, with AF included as a reference. The computational data for snapping energies, bond dissociation energies (BDEs), bond dissociation free energies (BDFEs), and aquation free energies (ΔG_aq_) provide valuable insights into the stability of the Au–ligand bonds in AF, AF-Cl, and RDL-15 (Figure 4). These findings can help interpret the observed experimental results, specifically the differences in cytotoxicity and inhibitory activity. The results suggest that the stability of the Au–ligand bond is a key factor influencing the biological activity of these compounds.

**Figure 4:**
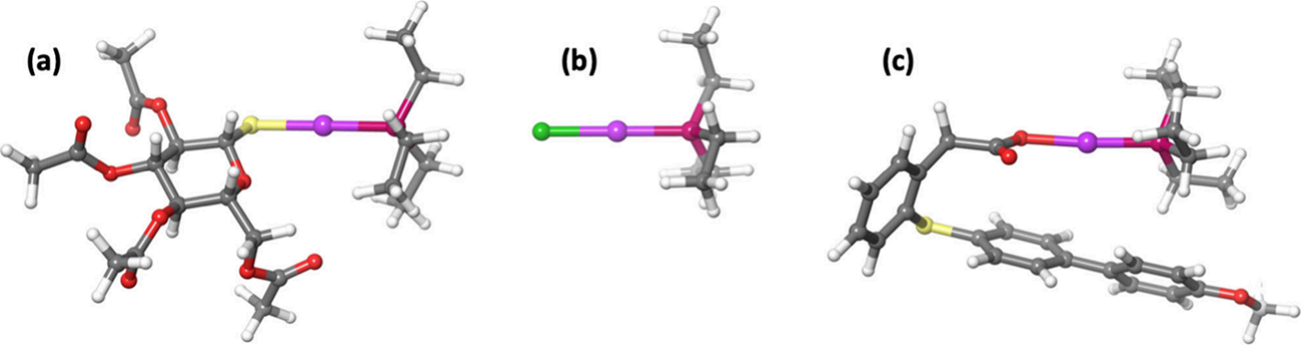
3D structures of the most stable conformers of AF (a), AFCl (b), and RDL-15 (c). Color scheme: Au (plum), Cl (green), S (yellow), O (red), N (blue), C (gray), and H (white).

The calculated bond strengths for the Au–P bond follow a clear trend: RDL-15 (80.1 kcal/mol), AF-Cl (71.1 kcal/mol), and AF (64.0 kcal/mol). This trend is also reflected in the BDEs and BDFEs, with RDL-15 having the highest values for both (BDE: 63.9 kcal/mol, BDFE: 52.1 kcal/mol) (Table 3). The Mulliken charge analysis supports this finding, showing that the PEt_3_ fragment in RDL-15 has the highest positive charge (0.43), suggesting a stronger electron donation to the gold center, which in turn strengthens the Au–P bond (Table 4). The strength of the other gold–ligand bond (Au–S, Au–Cl, or Au–O) is also important. The Au–Cl bond in AF-Cl has the lowest snapping energy (39.3 kcal/mol), BDE (38.4 kcal/mol), BDFE (30.6 kcal/mol), and ΔG_aq_ (22.0 kcal/mol) (Table 3). This suggests that the Au–Cl bond is the weakest of the three, making it more labile under physiological conditions. The Au–O bond in RDL-15 is comparably stronger than the Au–Cl bond, with a snapping energy of 52.6 kcal/mol, BDE of 47.1 kcal/mol, and BDFE of 33.3 kcal/mol (Table 3). Importantly, the calculated ΔG_aq_ for RDL-15 (26.3 kcal/mol) is higher than that of the known active species AF-Cl, indicating that the Au–O bond is thermodynamically more robust toward spontaneous aquation than the chloride counterpart. These values indicate a significant intrinsic reactivity, yet the high endergonicity (>20 kcal/mol) for the detachment of the labile ligand suggests that RDL-15 remains sufficiently stable in bulk solution during the timeframes of the biological assays performed. This provides a molecular basis for the controlled release of the pharmacophores, which, while appearing rapid under high-concentration NMR conditions, is thermodynamically governed to ensure availability of the intact species at the lower concentrations relevant to the biological milieu. On the other hand, we envision that the decomplexation of the RDL-15 ligand could be further promoted by the protonation of the carboxylate moiety occurring in the acidic low pH context, e.g. the tumor microenvironment, or through assisted mechanisms within the protein binding pockets, where residues may provide hydrogen bonding to lower the activation barrier. This is a key difference between RDL-15 and AF-Cl, and it aligns with the experimental finding that AF-Cl shows similar cytotoxicity on ovarian cell lines compared to RDL-15. While a higher BDFE usually implies greater thermodynamic stability, a weaker Au–ligand bond could facilitate the release of the cytotoxic gold­(I) center, leading to higher activity. The Au–S bond in auranofin is the strongest of the three, with a snapping energy of 55.6 kcal/mol, BDE of 51.6 kcal/mol, and BDFE of 39.8 kcal/mol (Table 3). The Mulliken charge on the thioglucose/Cl/LP-158 fragment is most negative for RDL-15 (−0.62), followed by AF-Cl (−0.60), and least negative for auranofin (−0.47) (Table 4). This increased electron density on the ligand in RDL-15 corroborates the possible assistance by protonation to the Au–O bond dissociation compared to the Au–Cl bond in AF-Cl, which may explain why RDL-15 shows greater inhibition of cell migration/invasion, a property that is likely related to its favored decomplexation in the tumor microenvironment.

**Table 3:** Snapping Energies, BDEs, BDFEs, and Aquation Free Energies (ΔG_aq_) of AF, AF-Cl, and RDL-15^a^

Compound	Bond	Snapping energy	BDE	BDFE	ΔG_aq_
AF	Au–P	64.0	58.1	44.9	36.4
	Au–S	55.6	51.6	39.8	32.7
AF-Cl	Au–P	71.1	66.4	55.4	43.6
	Au–Cl	39.3	38.4	30.6	22.0
RDL-15	Au–P	80.1	63.9	52.1	45.1
	Au–O	52.6	47.1	33.3	26.3

a All values are reported in kcal/mol.

**Table 4:** Mulliken Charge Distribution in AF, AF- Cl, and RDL-15

	Mulliken charges		
Fragment or Atom	AF	AF-Cl	RDL-15
Au	0.10	0.22	0.19
P	0.13	0.09	0.16
PEt_3_	0.36	0.37	0.43
S/Cl/O	–0.51	–0.60	–0.57
Thioglucose/Cl/LP-158 (deprotonated)	–0.47	–0.60	–0.62

In conclusion, this work addresses key biological features that underline the aggressive and therapy-refractory nature of ovarian cancer by integrating redox dysregulation and extracellular matrix remodeling within a single molecular strategy. OC progression and adverse clinical outcome depend not only on sustained tumor cell survival, but also on early dissemination and tissue invasion. On this basis, RDL-15 was conceived as a modular gold­(I) prodrug that integrates the auranofin-derived Au–phosphine pharmacophore with the MMP-2/9 inhibitor LP-158. The compound maintains the cytotoxic properties of the gold­(I) core in all ovarian cancer models tested, including platinum-resistant cells. At the same time, it acquires the capacity to modulate processes linked to migration and invasion. TrxR inhibition was consistently observed across different cellular contexts, indicating persistent targeting of redox-regulated pathways. In parallel, enzymatic and functional analyses show that metalloproteinase blockade primarily affects invasive traits, with minimal impact on cell viability. Computational analysis of Au–ligand interactions provide a consistent molecular interpretation for the observed biological profile, rationalizing the complex’s intrinsic reactivity and its enhanced activity in the tumor microenvironment. Specifically, the assisted decomplexation mechanism facilitated by the target environment justifies the dual-action efficiency despite the thermodynamic robustness of the Au–O bond. The finding that RDL-15 sits in a stability window between AF and AF-Cl, being even more resistant to spontaneous aquation than the latter, confirms its viability as a single chemical entity in aqueous media. Although RDL-15 does not display substantially greater cytotoxic potency than the combination of its parent compounds, its covalent design provides distinct pharmacological advantages. Incorporation of both pharmacophores within a single chemical entity ensures fixed stoichiometry and coordinated intracellular exposure, thereby minimizing potential pharmacokinetic discrepancies associated with drug combinations. This structural integration enforces concurrent modulation of redox homeostasis and extracellular matrix remodeling within the same cellular context. The mechanistic inseparability of the two activities may also reduce the likelihood of adaptive resistance arising from differential regulation of individual targets. Together, these features substantiate the rationale for a single-entity dual-target strategy beyond simple additive effects. Given the promising results obtained in this study, future work will prioritize validating RDL-15 in more physiologically relevant 3D models-such as spheroid and patient-derived organoids-to better assess its translational potential before advancing to in vivo studies. Overall, our results strengthen the idea that dual-action metal-based prodrugs can address complementary weaknesses linked to tumor persistence and dissemination. This strategy offers a rational basis for developing next-generation therapies for aggressive and treatment-resistant OC.

## Supplementary Material



## Notes

Synthesis of RDL-15; NMR spectra of RDL-15; HRMS spectra of RDL-15; In-solution stability of RDL-15 including plasma studies; Log*P* evaluation; Enzymatic assay; Cellular studies (Cellular growth conditions and Cell viability assay); Thioredoxin reductase inhibition assay; Migration/invasion assay; Computational methods (PDF)

## Notes

No unexpected or unusually high safety hazards were encountered in the reported experiments. The Human plasma used in this study (^31^P NMR RDL-15 stability experiments) was purchased commercially (Sigma-Aldrich) and was fully anonymized, therefore, no institutional ethical approval was required.

## Abbreviations

AF: Auranofin
AF-Cl: Et_3_PAuCl
BDE: bond dissociation energy
BDFE: bond dissociation free energy
ECM: extracellular matrix
EOC: epithelial ovarian cancer
IC_50_: half-maximal inhibitory concentration
MMP: matrix metalloproteinase
MT1-MMP: membrane-type 1 matrix metalloproteinase
OC: ovarian cancer
TrxR: thioredoxin reductase
ΔG_aq_: aquation free energies
ESI-MS: electrospray ionization mass spectrometry
MTT: 3-(4,5-dimethylthiazol-2-yl)-2,5-diphenyltetrazolium bromide
SD: standard deviation
