# Supplementary material for: A Dual-Action Gold(I) Prodrug Targeting Redox Homeostasis and Extracellular Matrix Remodeling in Ovarian Cancer
Source: ACS Med Chem Lett. 2026 Jun 8;17(7):1555–62. doi: 10.1021/acsmedchemlett.6c00118 (PMC13358944; doi:10.1021/acsmedchemlett.6c00118)
Supplement: Supplementary file 1 [file ml6c00118_si_001.pdf]

SUPPORTING INFORMATION FOR

A Dual-Action Gold(I) Prodrug Targeting Redox Homeostasis and Extracellular Matrix Remodeling in Ovarian Cancer

Riccardo Di Leo,[1,2,*] Enrico Crispino,[1] Lorenzo Chiaverini,[1] Luca Famlonga,[1] Rosamaria Militello,[3] Tania Gamberi,[3] Iogann Tolbatov,[4] Alessandro Marrone,[5] Diego La Mendola,[1] Tiziano Marzo,[1,*] Elisa Nuti[1]

[1]*Department of Pharmacy, University of Pisa, via Bonanno 6, 56126 Pisa, Italy.*

[2]*Institute of Clinical Physiology, National Research Council (CNR), via Moruzzi 1, 56124 Pisa, Italy.*

[3]*Department of Experimental and Clinical Biomedical Sciences "Mario Serio", University of Florence, Viale GB Morgagni 50, 50134 Florence, Italy*

[4]*Department of Chemical, Physical, Mathematical and Natural Sciences, University of Sassari, 07100 Sassari, Italy*

[5]*Department of Pharmacy, University "G. D'Annunzio", via dei Vestini 31, 66100 Chieti, Italy.*

**Corresponding Authors: Riccardo Di Leo, email: riccardo.dileo@cnr.it;Tiziano Marzo, email: tiziano.marzo@unipi.it*

Table of Contents

Synthesis of RDL-15 S2

NMR spectra of RDL-15 S4

HRMS spectra of RDL-15 S5

In-Solution Stability of RDL-15 S6

Log*P* Evaluation S7

Enzymatic assay S7

Cellular Studies S8

Thioredoxin reductase inhibition assay S9

Migration/invasion assay S9

Computational methods S10

General synthetic methods

All reagents and solvents were obtained from commercial suppliers and employed as received unless otherwise stated. Auranofin was sourced from Cayman Chemical Company. All synthetic procedures involving gold complexes were conducted under an inert nitrogen atmosphere using conventional Schlenk techniques. The isolated compounds were stored at −20 °C and protected from light. ^{1}H, ^{13}C, and ^{31}P NMR spectra were acquired on a Bruker Avance II 400 spectrometer operating at 400.0 MHz for ^{1}H, 100.6 MHz for ^{13}C, and 162.0 MHz for ^{31}P. Chemical shifts (δ) are reported in parts per million (ppm) and referenced to the residual signals of the corresponding deuterated solvents. Spectral processing was performed using MestreNova software (version 14.2.3). Elemental analyses (C, H, N) were performed using an EMASoft elemental analyzer (VELP Scientifica). High-resolution electrospray ionization mass spectra (HR-ESI-MS) were collected on an Orbitrap mass spectrometer (Thermo Scientific, San Jose, CA, USA) equipped with a heated electrospray ionization (HESI) source. UV–visible absorption spectra were recorded on an Agilent Cary 60 UV–Vis spectrophotometer using quartz cuvettes with a 1 cm optical path length.

Synthesis of RDL-15

Synthesis of argentum-2-(2-((4'-methoxy-[1,1'-biphenyl]-4-yl)thio)phenyl)acetate (**Ag^{+} - LP-158**)

An aqueous suspension of the carboxylic acid LP-158 (150 mg, 0.428 mmol, 1.0 equiv) was treated with NaOH (4 mL of solution) and stirred at RT for 10 min to ensure complete deprotonation. Silver nitrate ($AgNO_3$, 94.5 mg, 0.556 mmol, 1.3 equiv) was then added, and the resulting mixture was kept under light-protected conditions while stirring at RT for an additional 50 min. During this time, the formation of a brown precipitate was observed. The solid product was isolated by filtration through a Celite pad and thoroughly washed with water, followed by ethanol. Yield: 91%.

Synthesis of 2-(2-((4'-methoxy-[1,1'-biphenyl]-4-yl)thio)phenyl)-1-(η^1-oxidaneyl)ethan-1-one-O-(triethyl-phosphine)-gold(I)] (**RDL-15**)

Et_3PAuCl (135 mg, 0.387 mmol, 1 equiv.) was dissolved in ethanol (3 mL) in a Schlenk flask under an inert atmosphere. The sodium salt of LP-158 (Na^{+}-LP-158, 177mg, 0.387 mmol, 1 equiv.) was added, and the reaction mixture was stirred at RT for 3 h under light-protected conditions. Upon completion, the suspension was filtered through a celite pad, and the solvent was removed under reduced pressure. The resulting oil was triturated with Et_2O/CH_2Cl_2 to afford the target compound RDL-15 as a hygroscopic brown solid (77 mg).

Yield: 30%. ^{1}H NMR (400 MHz, DMSO-d_6) δ: 7.59-7-55 (m, 4H); 7.33-7.21 (m, 6H); 7.01-6.99 (m, 2H); 3.78 (s, 1H); 3.58 (s, 2H); 1.86 (t, *J* = 8.8 Hz, 6H); 1.09-1.01 (m, 9H). ^{13}C NMR (100 MHz, DMSO-d_6) δ: 159.0; 156.9; 149.8; 148:6; 140.2; 138.0 133.3;131.6; 131.2; 129.8; 128.0; 127.6; 126.9; 114.4; 55.2; 31.7; 16.7 (d, *J* = 38 Hz); 8.9. ^{31}P NMR (162 MHz, DMSO-d_6) δ: 27.51. HRMS (ESI, *m/z*) calculated for $C_{27}H_{33}AuO_3PS$ $[M+H]^+$: 665.15308; found: 665.08240; calculated for $C_{27}H_{32}AuO_3PSCl$ $[M-Cl]^-$: 699.11693; found: 699.11835. Elemental analysis for $C_{27}H_{32}AuO_3PS{\cdot}1CH_2Cl_2$, calculated: % C, 44.87; % H, 4.57; Found: % C, 45.05; % H, 4.70.

NMR spectra of RDL-15

^1H NMR (400 MHz, DMSO-*d*6)

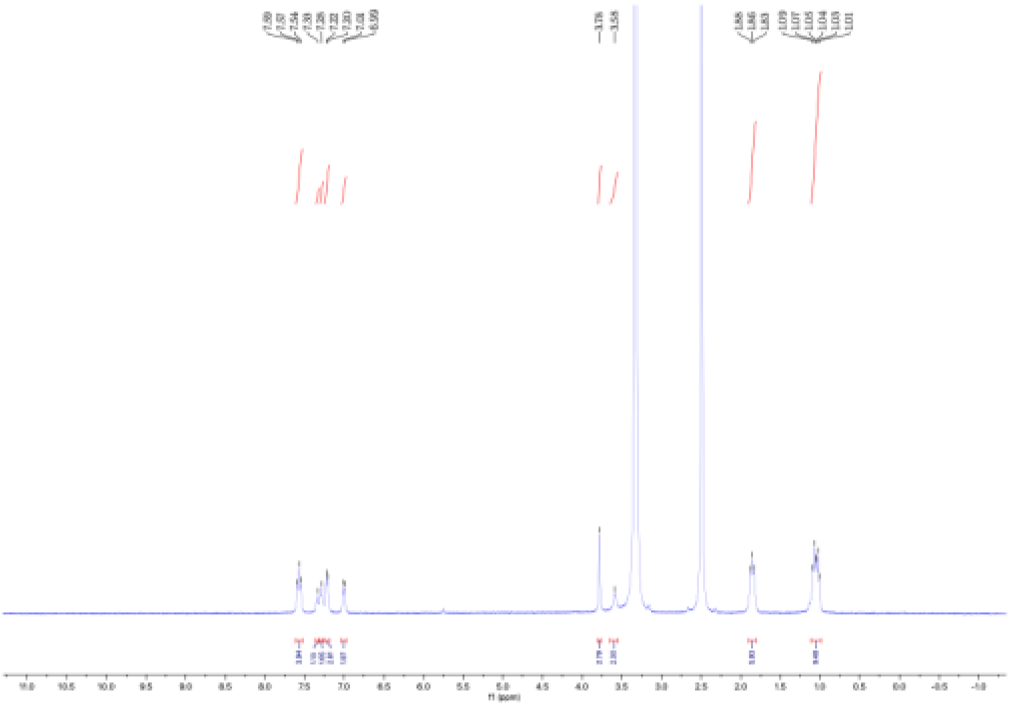

^{13}C NMR (100 MHz, DMSO-*d*6)

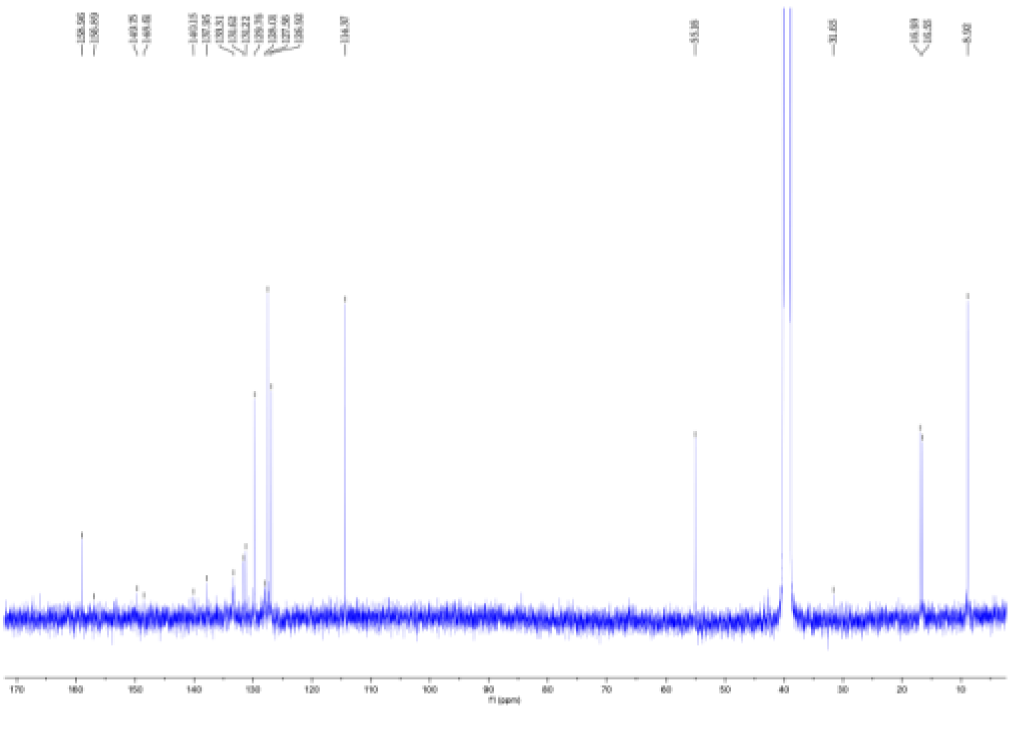

^{31}P NMR (162 MHz, DMSO-d_6)

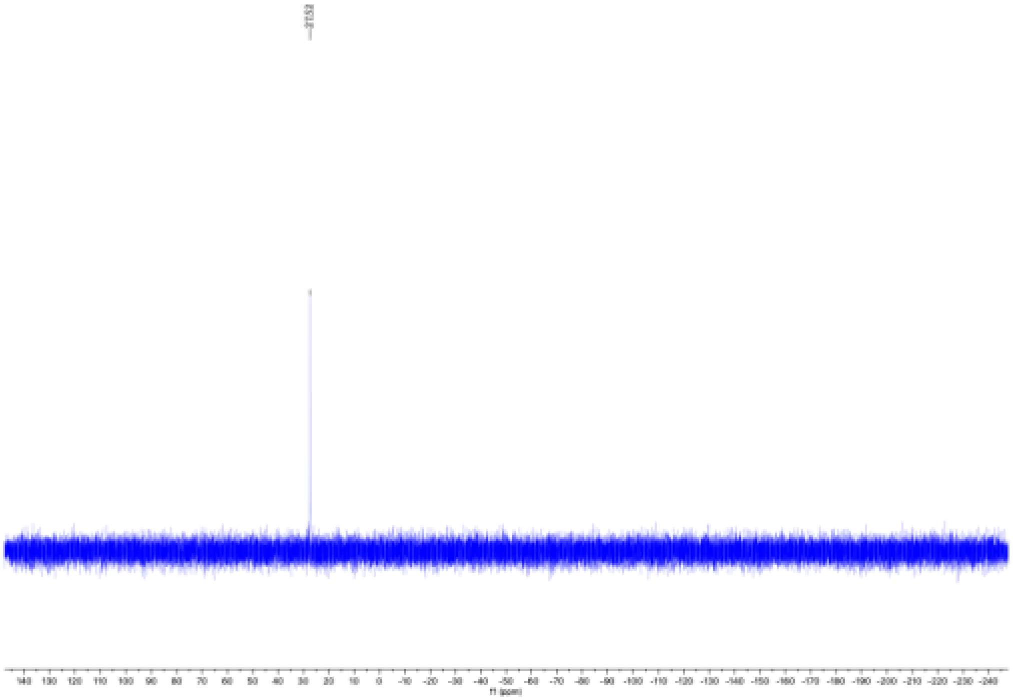

HRMS spectrum of compound RDL-15

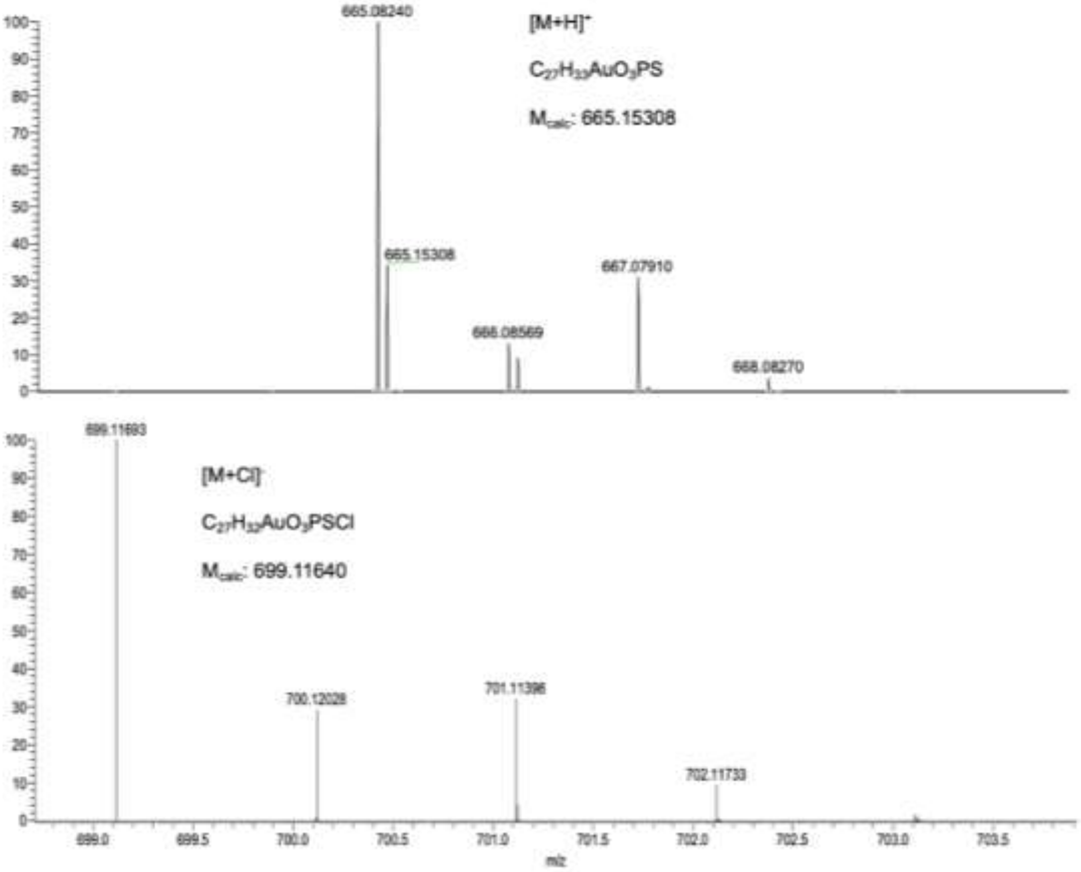

In-Solution Stability of RDL-15

The solution stability of RDL-15 was investigated by complementary ^{31}P NMR and UV–Vis absorption spectroscopy under aqueous conditions. ^{31}P NMR experiments were performed at 37°C in a DMSO/D_2O mixture (1:1, v/v) at a final concentration of 4.3 × 10^{-3} M. Spectra were recorded immediately after sample preparation and at increasing incubation times over 48 h. Plasma stability was assessed by NMR spectroscopy using a plasma solution of the complex (5.4 × 10^{-3} M), prepared according to the procedure of Berners-Price and Sadler in the presence of 5% DMSO and 25% D_2O.[1]

UV–Vis measurements were carried out under comparable conditions in a DMSO/PBS mixture (1:1, v/v), at a concentration of 1.0 × 10^{-4} M. Absorption spectra were collected in the 200–500 nm wavelength range at selected time points over a 48 h incubation period at 37°C.

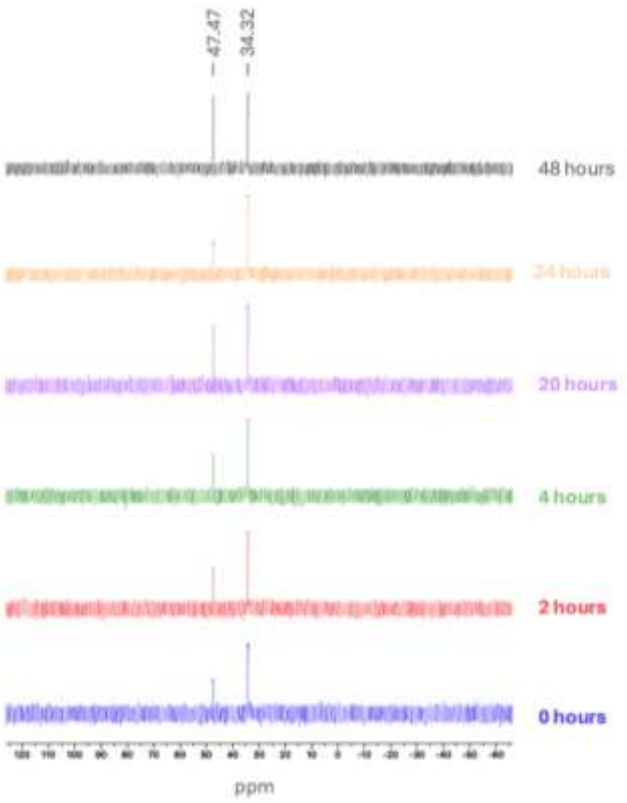

Figure S1. ^{31}P NMR spectra of RDL-15 (37°C, solvent DMSO/D_2O 1:1) recorded at different time intervals, showing the time-dependent evolution of the solution composition.

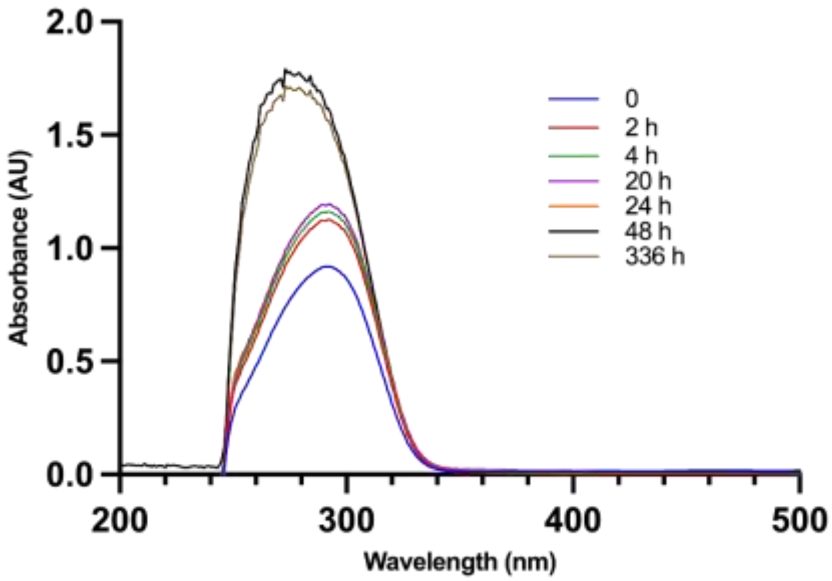

Figure S2. UV-Vis spectra of RDL-15 (37°C, solvent DMSO:PBS 1:1) recorded at different time intervals, showing the time-dependent evolution of the solution composition.

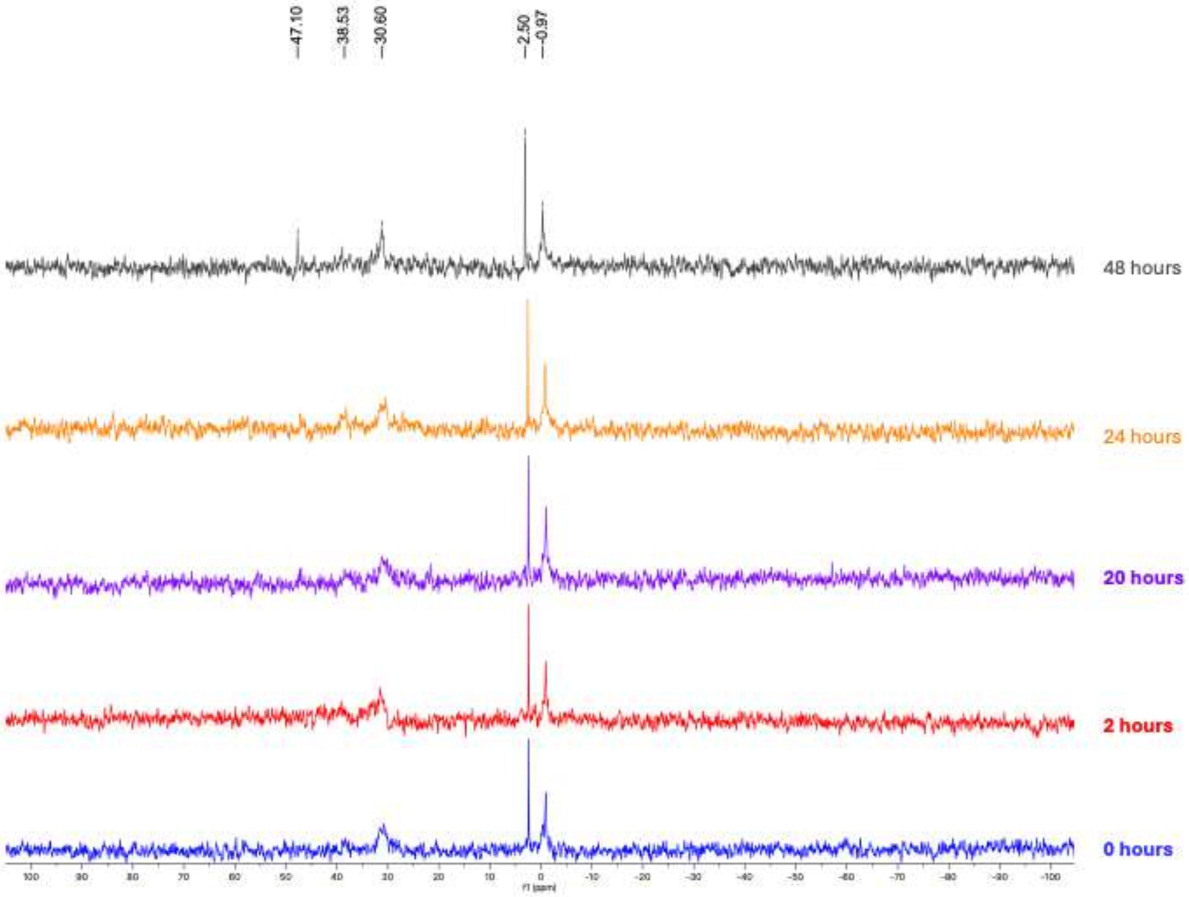

Figure S3. ^{31}P NMR spectra of RDL-15 (37°C, in presence plasma with 5 % DMSO) recorded at increasing time intervals. The signals at 2.50 and 0.97 ppm are consistent with inorganic phosphate and phospholipid species, respectively.

Log*P* Evaluation

The partition coefficient was evaluated using a shake–flask approach combined with UV–visible spectrophotometric analysis, following established protocols reported in the literature. All measurements were conducted at RT. A concentrated stock solution of RDL-15 was prepared in octanol pre-equilibrated with water. Quantitative UV–Vis analysis was performed at the absorption maximum of the compound (λ = 288 nm). The experiment was repeated three times using the same stock solution, and the resulting Log*P* value is reported as the mean ± standard deviation.

Enzymatic assay

Pro-MMP-2 (PF037) and pro-MMP-9 (PF038), were purchased from Calbiochem (Merck-Millipore). Proenzymes were activated immediately prior to use with p-aminophenylmercuric acetate (APMA 2 mM for 1 h at 37°C for MMP-2, 1 mM for 1 h at 37°C for MMP-9). For assay measurements, each inhibitor stock solution (DMSO, 10 mM) was further diluted in the fluorometric assay buffer (FAB: Tris 50 mM, pH = 7.5, NaCl 150 mM, CaCl2 10 mM, Brij 35 0.05% and DMSO 1%) at seven different concentrations. Activated enzyme (final concentration 0.56 nM for MMP-2 and 1.3 nM for MMP-9) and inhibitor solutions were incubated in FAB for 3 h at 25 °C. After the addition of 200 μM solution of the fluorogenic substrate Mca-Lys-Pro-Leu-Gly-Leu-Dap(Dnp)-Ala-Arg-NH_2 (Bachem) for all the enzymes in DMSO (final concentration 2 μM for all enzymes), the hydrolysis was monitored every 10 sec. for 15 min. recording the increase in fluorescence (λex = 325 nm, λem = 400 nm) using a SpectraMax Gemini XPS (Molecular Devices, Sunnyvale, CA) plate reader. The assays were performed in duplicate in a total volume of 200 μL per well in 96-well microtitre plates (Corning, black, NBS). Control wells lack inhibitor. The MMP inhibition activity was expressed in relative fluorescent units (RFU). Percent of inhibition was calculated from control reactions without the inhibitor. IC_{50} was determined using the formula: $v_i/v_o = 1/(1 + [I]/ IC_{50})$, where v_i is the initial velocity of substrate cleavage in the presence of the inhibitor at concentration [I] and v_o is the initial velocity in the absence of the inhibitor. Results were analyzed using SoftMax Pro software (version 5.4.3, Molecular Devices, Sunnyvale, CA) and Prism Software version 5.0 (GraphPad Software, Inc., La Jolla, CA, USA).

Cellular Studies

Cell culture medium RPMI, fetal bovine serum (FBS), and phosphate-buffered saline were obtained from Euroclone (Milan, Italy). General chemicals used for the biological experiments were purchased from Merck, unless otherwise indicated.

Cellular growth conditions

The A2780 human ovarian carcinoma cell line (Lot No. CSC-C9491J) and A2780/R resistant to cisplatin (Lot No. CSC-C9492J) were sourced from Creative Bioarray, while the SKOV-3 line was obtained from Cell Line Service GmbH (300342-1420SF). A2780/AF-R cells were generated by continuously exposing the parental A2780-sensitive line to gradually increasing concentrations of auranofin. The process began by culturing the parental cells in standard medium containing a low, sublethal dose of auranofin (100 nM). After 3–4 passages under these conditions, the AF concentration was raised stepwise on six successive occasions until reaching a final concentration of 7 µM. This level of resistance was established over an 8-month selection period. The ovarian cancer cells were maintained in RPMI 1640 medium containing 10% FBS, 1% glutamine, and 1% antibiotics, incubated at 37°C and 5% CO2 humidified atmosphere. These cultures were passaged twice a week with a 1:5 split ratio (aiming for 3–6×10^4 cells/mL). Human embryonic kidney cells HEK-293 was kindly provided by Prof. Magherini. Cell lines were maintained in DMEM supplemented with 10% FBS, 1% glutamine, and 1% antibiotics, at 37°C and 5% CO2 humidified atmosphere. All cell cultures were passed twice a week with a 1:5 split ratio (aiming for 3–6×10^4 cells/mL) and underwent regular mycoplasma contamination screening.

Cell viability assay

The antiproliferative effects of the compounds were assessed using the MTT [3-(4,5-dimethylthiazol-2-yl)-2,5-diphenyltetrazolium bromide] assay. Briefly, A2780/S, A2780/R, A2780/AF-R and SKOV-3 cells were seeded (1×10^4 cells/well) in 96-well microplates and allowed to adhere for 24 hours. HEK293 cells were seeded at 8×10^4 cells/well under the same conditions. Subsequently, cells were exposed to varying concentrations (ranging from 0.003 to 100 µM) of gold complexes for a 72-hour incubation period at 37 °C in a humidified atmosphere. Following the 72-hour treatment, the medium was replaced with 0.5 mg/mL MTT solution, and cells were incubated for an additional hour at 37 °C. The resulting formazan crystals were solubilized in DMSO, and the absorbance was measured at 595 nm

using a BioRad microplate reader equipped with Microplate Manager/PV software (v. 4.0). The half-maximal inhibitory concentration (IC_{50}) was determined from the absorbance data using GraphPad Prism v 6.0 (Graphpad Holdings, LLC, USA) using a non-linear regression analysis with a four-parameter logistic (4PL) dose–response model. The Top and Bottom parameters were not constrained, the HillSlope was allowed to vary, and the X-axis was set to $\log_{10}$ concentration. All experiments were conducted in triplicate to ensure reproducibility.

Thioredoxin reductase inhibition assay

Thioredoxin reductase (TrxR) activity was measured using a colorimetric kit (Sigma-Aldrich CS0170), which quantifies the NADPH-dependent reduction of DTNB to TNB at 412 nm. Because other cellular enzymes can also reduce DTNB, the assay includes a selective TrxR inhibitor to determine TrxR-specific activity. A2780 and SKOV3 cells were treated for 24 h with concentrations equivalent to their 72 h IC_{50} values, then lysed in RIPA buffer (50 mM Tris–HCl pH 7.0, 1% NP-40, 150 mM NaCl, 2 mM EGTA, 100 mM NaF) supplemented with protease inhibitors. Protein content was quantified by Bradford assay, and 30 µg of lysate protein were used per reaction. Activities were normalized to total protein.

Experiments were performed in triplicate (three independent biological replicates). Statistical analysis was conducted using one-way ANOVA followed by Tukey's test (GraphPad Prism v 6.0), with $p < 0.05$ considered significant.

Migration/invasion assay

Migration/invasion was assessed using a Boyden chamber equipped with 8 µm pore filters (Corning) coated with 50 µg/cm² Matrigel (Corning). A total of 3×10^4 SKOV3 cells, serum-deprived overnight, were seeded into the upper chamber in serum-free medium with or without compounds at concentrations corresponding to their 72 h IC_{50} values. Complete medium containing 10% FBS was added to the lower chamber as a chemoattractant. After 16 hours, migrated cells were quantified. Non-migrated cells were removed from the upper surface of the membrane, while cells on the lower surface were stained using the Diff-Quik Staining Kit according to the manufacturer's instructions. Migrated cells were counted in at least 5 random fields of the lower membrane surface. Experiments were performed in triplicate (three independent biological replicates). Statistical analysis was conducted using one-way ANOVA followed by Tukey's test (GraphPad Prism v 6.0), with $p < 0.05$ considered significant.

Computational methods

A thorough computational analysis was performed to characterize the structures and bond strengths of the metal complexes. For the initial conformational search, the CREST software package was utilized.[2] This tool efficiently explores the torsional and rotameric space by combining an effective search algorithm with a tight-binding density functional theory approach xTB for estimating electronic energies. These calculations were performed in water to mimic a biological environment.

All obtained conformers were optimized using the ωB97X-D range-corrected hybrid density functional.[3] This functional was chosen for its proven accuracy in determining geometries and reaction profiles for compounds containing transition metals.[4–6] Initial optimizations utilized the def2SVP basis set,[7] with the most stable conformers being subsequently refined with the more accurate def2TZVP basis set.[7] This two-step approach ensures a balance between computational efficiency and accuracy. The def2TZVP basis set was also used for the final single-point electronic and solvation energy calculations. All quantum chemistry computations were carried out using the Gaussian 16 C.01 software package.[8] Density functional theory (DFT) is a widely used and effective method for characterizing the structures of complexes including gold.[9-11]

The effects of an aqueous environment were modeled using the IEFPCM (Integral Equation Formalism Polarizable Continuum Model) continuum solvent method.[12] This model is particularly effective for calculating aqueous free energies of solvation for charged and neutral species, providing a precise assessment of solution properties.[13] Frequency calculations were also performed to confirm that the optimized structures were true stationary points and to derive zero-point energy and thermal corrections.

The strength of the Au-ligand bonds was quantified by calculating snapping energies, bond dissociation enthalpies (BDEs), and bond dissociation free energies (BDFEs). Snapping energies, which represent the energy required to break a bond into unrelaxed fragments, were determined by subtracting the single-point electronic energies of the two fragments at the complex's geometry from the electronic energy of the full, optimized complex. In contrast, BDEs and BDFEs account for the energy of both the full complex and the individual fragments after they have fully relaxed. BDEs represent the difference in enthalpy, while

BDFEs represent the difference in free energy between the products and reactants. This comprehensive approach provides a detailed and multi-faceted view of bond stability.

The thermodynamic barrier to spontaneous ligand exchange was further evaluated by calculating the aquation free energy (ΔG_{aq}), modeled as the displacement of the labile ligand by a water molecule to form the $[Et_3PAu(H_2O)]^+$ aqua-adduct. These calculations included standard state corrections from 1 atm to 1 M to ensure accuracy in the aqueous phase. Additionally, Mulliken population analysis was performed on optimized geometries to determine the electronic distribution across the gold center and coordinating atoms. This electronic characterization provides a basis for rationalizing observed bond strengths and the potential for protonation-assisted decomplexation within the acidic tumor microenvironment.

References

(1) Berners-Price, S. Interaction of the Antitumor Au(I) Complex [Au(Ph2P(CH2)2PPh2)2]Cl with Human Blood Plasma, Red Cells, and Lipoproteins: 31P An. *Journal of Inorganic Biochemistry* **1987**, *31* (4), 267–281. https://doi.org/10.1016/0162-0134(87)80081-8.

(2) Pracht, P.; Bohle, F.; Grimme, S. Automated Exploration of the Low-Energy Chemical Space with Fast Quantum Chemical Methods. *Phys. Chem. Chem. Phys.* **2020**, *22* (14), 7169–7192. https://doi.org/10.1039/C9CP06869D.

(3) Chai, J.-D.; Head-Gordon, M. Long-Range Corrected Hybrid Density Functionals with Damped Atom–Atom Dispersion Corrections. *Phys. Chem. Chem. Phys.* **2008**, *10* (44), 6615. https://doi.org/10.1039/b810189b.

(4) Tolbatov, I.; Marzo, T.; Umari, P.; La Mendola, D.; Marrone, A. Detailed Mechanism of a DNA/RNA Nucleobase Substituting Bridging Ligand in Diruthenium(II , III) and Dirhodium(II , II) Tetraacetato Paddlewheel Complexes: Protonation of the Leaving Acetate Is Crucial. *Dalton Trans.* **2025**, *54* (2), 662–673. https://doi.org/10.1039/D4DT02621G.

(5) Tolbatov, I.; Umari, P.; Marrone, A. The Binding of Diruthenium (II,III) and Dirhodium (II,II) Paddlewheel Complexes at DNA/RNA Nucleobases: Computational Evidences of an Appreciable Selectivity toward the AU Base Pairs. *Journal of Molecular Graphics and Modelling* **2024**, *131*, 108806. https://doi.org/10.1016/j.jmgm.2024.108806.

(6) Tolbatov, I.; Cirri, D.; Tarchi, M.; Marzo, T.; Coletti, C.; Marrone, A.; Messori, L.; Re, N.; Massai, L. Reactions of Arsenoplatin-1 with Protein Targets: A Combined Experimental and Theoretical Study. *Inorg. Chem.* **2022**, *61* (7), 3240–3248. https://doi.org/10.1021/acs.inorgchem.1c03732.

(7) Weigend, F.; Ahlrichs, R. Balanced Basis Sets of Split Valence, Triple Zeta Valence and Quadruple Zeta Valence Quality for H to Rn: Design and Assessment of Accuracy. *Phys. Chem. Chem. Phys.* **2005**, *7* (18), 3297. https://doi.org/10.1039/b508541a.

(8) Frisch, M. J.; Trucks, G. W.; Schlegel, H. B.; Scuseria, G. E.; Robb, M. A.; Cheeseman, J. R.; Scalmani, G.; Barone, V.; Petersson, G. A.; Nakatsuji, H.; Li, X.; Caricato, M.; Marenich, A. V.; Bloino, J.; Janesko, B. G.; Gomperts, R.; Mennucci, B.; Hratchian, H. P.; Ortiz, J. V.; Izmaylov, A. F.; Sonnenberg, J. L.; Williams, Ding, F.; Lipparini, F.; Egidi, F.; Goings, J.; Peng, B.; Petrone, A.; Henderson, T.; Ranasinghe, D.; Zakrzewski, V. G.; Gao, J.; Rega, N.; Zheng, G.; Liang, W.; Hada, M.; Ehara, M.; Toyota, K.; Fukuda, R.; Hasegawa, J.; Ishida, M.; Nakajima, T.; Honda, Y.; Kitao, O.; Nakai, H.; Vreven, T.; Throssell, K.; Montgomery, J. A., Jr.; Peralta, J. E.; Ogliaro, F.; Bearpark, M. J.; Heyd, J. J.; Brothers, E. N.; Kudin, K. N.; Staroverov, V. N.; Keith, T. A.; Kobayashi, R.; Normand, J.; Raghavachari, K.; Rendell, A. P.; Burant, J. C.; Iyengar, S. S.; Tomasi, J.; Cossi, M.; Millam, J. M.; Klene, M.; Adamo, C.; Cammi, R.; Ochterski, J. W.; Martin, R. L.; Morokuma, K.; Farkas, O.; Foresman, J. B.; Fox, D. J. *Gaussian 16, Revision C.01*; Gaussian, Inc.: Wallingford, CT, **2016**.

(9) Chiaverini, L.; Pratesi, A.; Cirri, D.; Nardinocchi, A.; Tolbatov, I.; Marrone, A.; Di Luca, M.; Marzo, T.; La Mendola, D. Anti-Staphylococcal Activity of the Auranofin Analogue Bearing Acetylcysteine in Place of the Thiosugar: An Experimental and Theoretical Investigation. *Molecules* **2022**, *27* (8), 2578. https://doi.org/10.3390/molecules27082578.

(10) Cirri, D.; Marzo, T.; Tolbatov, I.; Marrone, A.; Saladini, F.; Vicenti, I.; Dragoni, F.; Boccuto, A.; Messori, L. In Vitro Anti-SARS-CoV-2 Activity of Selected Metal Compounds and Potential Molecular Basis for Their Actions Based on Computational Study. *Biomolecules* **2021**, *11* (12), 1858. https://doi.org/10.3390/biom11121858.

(11) Tolbatov, I.; Re, N.; Coletti, C.; Marrone, A. An Insight on the Gold(I) Affinity of *golB* Protein via Multilevel Computational Approaches. *Inorg. Chem.* **2019**, *58* (16), 11091–11099. https://doi.org/10.1021/acs.inorgchem.9b01604.

(12) Barone, V.; Cossi, M.; Tomasi, J. A New Definition of Cavities for the Computation of Solvation Free Energies by the Polarizable Continuum Model. *The Journal of Chemical Physics* **1997**, *107* (8), 3210–3221. https://doi.org/10.1063/1.474671.

(13) Klamt, A.; Moya, C.; Palomar, J. A Comprehensive Comparison of the IEFPCM and SS(V)PE Continuum Solvation Methods with the COSMO Approach. *J. Chem. Theory Comput.* **2015**, *11* (9), 4220–4225. https://doi.org/10.1021/acs.jctc.5b00601.
